# Study on the Morphological Development Timeline and Growth Model of Embryos and Larvae of European Catfish (*Silurus glanis*)

**DOI:** 10.3390/ani15172478

**Published:** 2025-08-23

**Authors:** Zhuoleaersi Adakebaike, Zhengwei Wang, Hudelati Anasi, Jiangtao He, Xuejie Zhai, Chunming Shi, Zhulan Nie

**Affiliations:** 1 College of Life Science and Technology, Tarim University, Alar 843300, China; zhuoleaersi@163.com (Z.A.); 10757232145@stumail.taru.edu.cn (Z.W.); 2 Xinjiang Production & Construction Corps Key Laboratory of Protection and Utilization of Biological Resources in Tarim Basin, Alar 843300, China; 3 Xinjiang Aquatic Science Research Institute/Northwest Fisheries Resources and Environment Scientific Observation and Experiment Station of the Ministry of Agriculture, Urumqi 830000, China; hudrat@163.com (H.A.); 1566193923@163.com (J.H.); xuejiezhai@foxmail.com (X.Z.); 13699955055@163.com (C.S.)

**Keywords:** *S. glanis*, embryonic development, larval–juvenile development

## Abstract

To systematically elucidate the chronological patterns of embryonic development and morphological changes in the larval and juvenile stages of *Silurus glanis*, and provide fundamental biological insights into this species, in this study, fertilized eggs were obtained through artificial spawning induction technology. After removing adhesiveness from fertilized eggs using trypsin, a detailed developmental study was conducted. The study systematically analyzed the chronological sequence of embryonic development and the morphological change patterns of larval and juvenile fish. The results showed the following: The fertilized eggs of *S. glanis* are yellow, spherical, and sticky, and the stickiness allows eggs to attach to spawning substrates, enhancing hatching success. The egg diameter after water absorption was (2.88 ± 0.13) mm. The embryonic development took 47 h and 55 min, with a total accumulated temperature of 1245.56 h degrees Celsius, the developmental process includes seven stages and twenty-six periods, namely the zygophase stage, cleavage stage, blastula stage, gastrula stage, neurula stage, organogenesis stage, and hatching stage. At a temperature of (26.0 ± 0.9) °C, the hatched individuals went through the pre-yolk sac larval stage, late larval stage, juvenile fry stage, and juvenile stage. In the pre-yolk sac larval stage, otoliths appeared in the bilateral otic vesicles, a pair of barbel primordia emerged under the mandible, a short and thin straight intestine formed in the abdominal cavity, and the oral fissure first appeared. In the late larval stage, the fin rays were initially formed, the intestine became thicker and longer, the oral fissure, anus, and cloaca were formed, and the larvae could float and start feeding on exogenous food. In the juvenile fry stage, the differentiation of various organs was basically complete, the nostrils became larger, and both the anal fin and caudal fin had dark black markings. In the juvenile stage, the maxillary barbels elongated, the mucus layer thickened on the body and back, the abdomen is light white, and it had the external morphological characteristics of an adult fish. By measuring and calculating the total length, body length, body height, and head length of *S. glanis* larvae and juveniles (0–40 days), the results showed that the growth characteristics conformed to the following fish growth formula: *TL* = 0.0141*x*^2^ + 0.8096*x* + 8.2421 (*R*^2^ = 0.9916), where x denotes days after hatching. This study has preliminarily mastered the chronological patterns of the embryonic development, growth, and formation of the morphological characteristics in larval and juvenile *S. glanis*, providing scientific data and laying a theoretical foundation for the division of early developmental stages, reproduction, hatching, and fry cultivation.

## Simple Summary

This study systematically examines the morphological development of *Silurus glanis* embryos and larval–juveniles. Fertilized eggs are yellow, sticky, spherical, and 2.88 ± 0.13 mm after water absorption. Embryonic development takes 47 h 55 min at (26.0 ± 0.9) °C, 1245.56 °C·h. Larval–juvenile development has four stages. A total length growth model (*TL* = 0.0141*x*^2^ + 0.8096*x* + 8.2421, *R*^2^ = 0.9916) supports early development research and aquaculture.

## 1. Introduction

Fish embryonic development, as the initial stage of ontogeny, is tightly regulated by environmental factors such as the temperature, dissolved oxygen, and water quality, while involving complex biological processes like cell differentiation, organogenesis, and functional maturation [1,2]. This process not only reveals the adaptive strategies of fish to natural environments but also provides a theoretical basis for optimizing artificial breeding techniques [3]. For instance, defining temperature thresholds for zebrafish embryonic development has improved hatching conditions and breeding efficiency [4], highlighting the significance of early development research in addressing the bottlenecks of seedling production and ensuring sustainable aquaculture amid global fishery resource decline and growing aquaculture demands.

Among numerous fish species, the *S. glanis*, as a typical representative of large carnivorous catfish, holds an important position in aquaculture due to its unique biological characteristics and considerable economic value. *S. glanis* belongs to the order Siluriformes, family Siluridae, and genus *Silurus* [5,6]. It has a smooth, scaleless body, dark coloration, a flattened head, and three pairs of barbels on the jaw, hence being commonly known as the “six-bearded catfish”. In the wild, this species is widely distributed in the river systems of the Baltic Sea, Black Sea, and Sea of Azov in central and eastern Europe, as well as the Caspian Sea and Aral Sea basins in western Asia [5]. As a favored economic fish in Europe, *S. glanis* is renowned as an “impeccable food fish” for its rapid growth, delicate meat, and few intermuscular spines [6]. With the increasing market demand, its artificial farming scale has been continuously expanding [6,7]. In terms of its introduction history, *S. glanis* was introduced to Lake Balkhash in Kazakhstan in the 1950s and successfully established itself [8], then further spread to the Yili River system in Xinjiang, China, via Central Asia, in the 1970s [7,9,10]. Initially, it mainly existed as a wild fish population. Since the 2000s, with the development of local aquaculture industry, it has gradually been incorporated into artificial breeding systems. Through domestication and cultivation, it has become a characteristic aquaculture species in Xinjiang, and large-scale farming has now been formed in regions such as the Yili River Valley and Bosten Lake [7,8].

In the development of *S. glanis* aquaculture, the scientific value of research on embryonic and larval–juvenile morphological development has become increasingly prominent. The embryonic and larval–juvenile stages are critical periods in a fish’s life history, with the developmental status of these stages directly determining the seedling survival rate, growth performance, and subsequent aquaculture benefits [9,10]. From an ecological perspective, the monitoring of fish eggs and fry serves as a core means to assess fish spawning ground scale, population dynamics, and resource recruitment [11,12]. Although progress has been made in research on the early development of some fish species, for example, the embryonic development timeline and larval growth characteristics of the European sea bass have been systematically elucidated [13], research on *S. glanis* in the field of embryonic development and larval growth remains significantly insufficient.

Current studies on *S. glanis* mostly focus on biological characteristics [6,8], capture and transportation [7], aquaculture techniques [14,15,16,17], salinity–alkalinity tolerance [18], and meat quality analysis [19,20], while the development mechanisms of its embryos and larval–juveniles remain unknown, urgently requiring systematic research to fill this critical gap in understanding. Given the unique ecological habits of *S. glanis* as a benthic predator, its embryonic development timing, larval–juvenile morphogenesis, and growth strategies may exhibit particularities. This paper aims to clarify the embryonic development timeline and accumulated temperature characteristics, reveal the allometric growth patterns and functional differentiation mechanisms of larval–juveniles, and establish growth prediction models to explore their practical applications in aquaculture. This research not only fills the gap in research on the early development of *S. glanis* but also provides key scientific bases for its artificial breeding, seedling cultivation, and resource protection.

## 2. Materials and Methods

### 2.1. Source of Broodstock and Artificial Propagation

In early April 2024, at the Aquatic Wildlife Rescue Center of Xinjiang, China, 12 sexually mature broodstock of the first fully artificially propagated generation of *S. glanis* were selected. The selected broodstock had a body length of 86.1 ± 9.2 cm, body weight of 4.57–8.39 kg, age of 5+ to 6+ years, and a female-to-male ratio of 1:1. These broodstock had exhibited natural reproductive behaviors such as chasing and pairing in the rearing pond, but due to the lack of ecological conditions required for natural reproduction in the pond, they were unable to complete natural spawning. Hence, artificial spawning induction technology was adopted to obtain fertilized eggs. The oxytocic drugs selected were luteinizing hormone-releasing hormone analog A2 (LHRH-A2), domperidone (DOM), and human chorionic gonadotropin (HCG). The oxytocic dosage for female fish was 5.0 μg/kg LHRH-A2, 2.0 mg/kg DOM, and 1000 IU/kg HCG, respectively, while male fish received half the female dosage. A two-injection method was adopted with a 24 h interval [21,22]. Injected broodstock were temporarily cultured in a greenhouse spawning pond (area: 57.8 m^2^, water depth: 1.2 m).

After being demucilaged with 5% trypsin solution, the fertilized eggs were incubated under conditions of a continuously controlled temperature ranging from 25.96 to 26.14 °C (maintained via HITOP HT-800 heaters (Hitop Industrial Technology Co., Ltd., Shenzhen, Guangdong, China; power: 500 W, temperature control accuracy: ±0.1 °C) to stabilize temperature), with dissolved oxygen ranging from 5.35 to 7.81 mg/L. Temperature was monitored every 2 h using a digital thermometer (precision: ±0.05 °C) to ensure stability throughout the incubation period. The pH value of the water was monitored daily at 9:00 a.m. throughout the experiment using a Leici PHS-3C precision pH meter (Shanghai INESA Scientific Instrument Co., Ltd., Shanghai, China; accuracy: ±0.01), maintained within the range of 7.3 ± 0.2. If the pH value was lower than 7.1, an appropriate amount of sodium bicarbonate (analytical grade) was added for adjustment; if it was higher than 7.5, dilute hydrochloric acid (1 mol/L) was used for fine-tuning. Thirty minutes after each adjustment, the pH value was remeasured to confirm its stability. We recorded abnormal situations during the experiment, including mortality, deformity, and developmental abnormalities. Throughout the experimental period, the mortality rate of embryos and larvae was less than 3%, and no obvious deformed individuals or random developmental deviations were observed. This might be related to the strictly controlled environmental conditions mentioned above, as the suitable environment reduced the occurrence of abnormal situations.

Artificial spawning induction was used to obtain fertilized eggs because preliminary observations confirmed that pond-reared broodstock cannot spawn naturally. Despite regulating the water temperature to 26.0 ± 0.9 °C and simulating natural substrate and light conditions, broodstock only exhibited chasing behavior without spawning, and no natural fertilized eggs were obtained in multiple pre-experiments. Thus, no natural spawning control group was included, and all experimental individuals originated from hormone-induced artificial propagation.

### 2.2. Observations on Embryonic Development and Larval–Juvenile Growth and Development

An SZN71 stereomicroscope (Ningbo Sunny Instruments Co., Ltd., Ningbo, Zhejiang, China; 10–40×) and a Nikon D5300 camera (Nikon Corporation, Tokyo, Japan; 24.2 megapixels; aperture: f/8; shutter speed: 1/100 s) were used, with over 30 embryos observed at each developmental stage (a stage was defined as when ≥50% of individuals reached that stage). Continuous observation was conducted for the first 2 h after fertilization, followed by observations every 0.5 h until hatching.

A T-series microscope (T2000; Olympus Corporation, Tokyo, Japan; 40–200×) and the same Nikon D5300 camera mentioned above were used to observe and photograph changes in the yolk sac of newly hatched larvae, as well as the morphological characteristics of larvae and juveniles during their growth and development. Observations were carried out daily from 1 to 20 days after hatching, and once every 5 days thereafter, with 10 individuals sampled each time.

For observations of live larvae and juveniles, anesthesia was performed using a 20 mg/L solution of tricaine methanesulfonate (MS-222) (Changsha Shanghe Biotechnology Co., Ltd., Changsha, Hunan, China) for 30–60 s until the individuals lost their motor response. Anesthetized individuals were observed and measured within 5 min to minimize physiological stress, after which they were transferred to fresh aerated water for recovery. The measured parameters included the total length, body length, head length, body height, and yolk sac diameter.

### 2.3. Data Processing and Analysis

Using a stage micrometer as a reference and image processing software (Adobe Photoshop CC 2015, Adobe Inc., San Jose, CA, USA), measurements were taken of the egg diameter of the fertilized eggs, as well as the total length, body length, head length, body height, and yolk sac diameter of larvae and juveniles. The obtained data were processed using Excel 2007 (Redmond, Washington, DC, USA), with results expressed as “mean ± standard deviation (mean ± SD)”. Adobe Photoshop CC was used to crop and arrange the images of embryonic development and larval–juvenile fish.

Due to constraints on broodstock availability and experimental conditions, this study was conducted as a single-batch observation. To ensure statistical robustness, statistical replication was achieved by expanding the sample size: for each stage of embryonic development, ≥30 individuals were observed (covering different egg clusters to reduce batch-specific bias); for growth measurements of larvae and juveniles, 10 individuals were randomly selected daily from different areas of the rearing tank (to minimize spatial heterogeneity within the population). Prior to analysis, all data were tested for normality using the Shapiro–Wilk test and for homoscedasticity using Levene’s test. Results are presented as mean ± standard deviation to reflect the central tendency and variability of the population.

#### 2.3.1. Accumulated Temperature Formula for Embryonic Development

The formula for calculating the accumulated temperature at each stage of embryonic development is as follows [23]:

*K* = *N* × *T*,

In the formula, *K* denotes the accumulated temperature (°C·h), *N* represents the time elapsed (h) from fertilization to a specific developmental stage, *T* is the average temperature (°C) during that developmental stage; the total accumulated temperature for the entire embryonic development process was calculated by summing the accumulated temperatures of each developmental stage, with the unit of °C·h.

#### 2.3.2. Yolk Sac Volume Formula

The yolk sac volume was calculated using the formula [24]:

*V* = 4/3π*R*/2 × (*r*/2)^2^

where *V* is the volume, *R* is the major axis, and *r* is the minor axis of the yolk sac.

#### 2.3.3. Assumptions and Rationale for Growth Model Selection

All growth model parameters in this study were calculated based on the population mean values. Ten individuals were randomly selected at each time point to measure indicators such as the total length, body height, and head length. By expanding the sample size (daily observations from 0–20 days post-hatching, and observations every 5 days from 20–40 days post-hatching) and controlling for spatial heterogeneity (random sampling from different aquaculture areas), we ensured that the data reflect population growth trends rather than individual differences. For model selection, we used the “Curve Estimation” function in SPSS Statistics 23.0. We initially fit 5 types of candidate models (linear, quadratic, cubic, exponential, logarithmic) to the relationship between days post-hatching (DAH, 0–40 days) and morphological traits (total length, body height, head length) of *S. glanis* larvae. Models were selected based on two criteria: statistical goodness of fit (coefficient of determination, *R*^2^) and biological relevance to early developmental stages. For total length (*TL*), the quadratic polynomial model (*TL* = 0.0141*x*^2^ + 0.8096*x* + 8.2421, *R*^2^ = 0.9916) outperformed the linear (*R*^2^ = 0.92, failed to capture post-20 DAH acceleration) and cubic (*R*^2^ = 0.93, overfitted early growth) models, as it could reflect the growth acceleration after 20 days post-hatching, which was consistent with the biological transition from endogenous (yolk sac) to exogenous feeding. For body height (BH), a fifth-order polynomial was chosen because lower-order models like linear (*R*^2^ = 0.82, missed multi-phasic dynamics) and cubic (*R*^2^ = 0.93, underestimated the 10–20 DAH slowdown) were insufficient. The fifth-order polynomial could depict its multi-phasic dynamics: rapid thickening during 1–10 days (driven by visceral organ development), a temporary slowdown during 10–20 days (prioritizing longitudinal growth), and secondary thickening during 20–40 days (as muscle and skeletal structures mature). For head length (*HL*), the cubic model (*HL* = 0.0002*x*^3^ + 0.0106*x*^2^ + 0.385*x* + 0.8476, *R*^2^ = 0.9840) was selected as it outperformed the linear model (*R*^2^ = 0.91, failed to reflect post-15 DAH slowdown). It could account for early rapid growth (1–15 days) driven by sensory organ (eye, barbel) and cranial bone development, followed by a gradual slowdown as the head proportion relative to the total length decreases.

## 3. Result

### 3.1. Embryonic Development Sequence and Main Characteristics of Fertilized Eggs

Mature eggs are yellow, spherical, and adhesive, with an average diameter of (2.88 ± 0.13) mm. Both water-absorbed fertilized eggs and mature eggs appeared light yellow briefly, and the perivitelline space expanded. After approximately 20 h, unfertilized eggs began to turn white, while fertilized eggs retained their original color. The accumulated temperature varied across different stages of *S. glanis* embryonic development, with the blastodisc stage having the lowest accumulated temperature and the organogenesis stage the highest (Table 1).

The embryonic development timeline and specific characteristics are as follows (Table 2):

#### 3.1.1. Zygote Stage (56 min–1 h 0 min)

**Figure 1:**
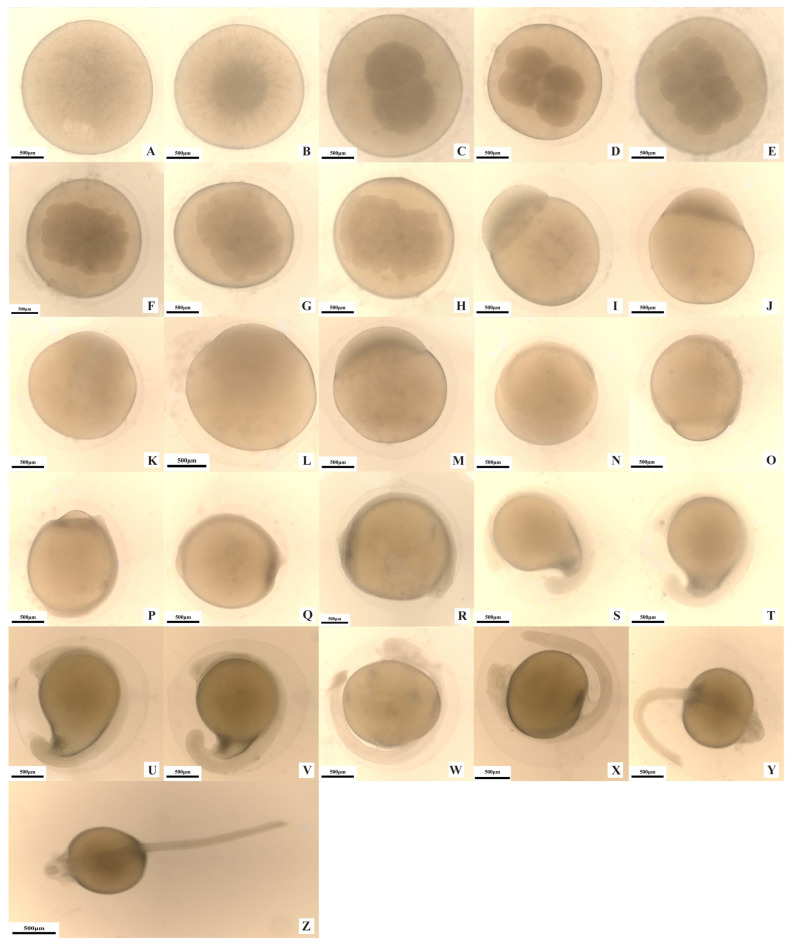
Embryonic development of *S. glanis* Linnaeus. (**A**) Fertilized egg; (**B**) blastodisc stage; (**C**) 2-cell stage; (**D**) 4-cell stage; (**E**) 8-cell stage; (**F**) 16-cell stage; (**G**) 32-cell stage; (**H**) 64-cell stage; (**I**) multicellular stage; (**J**) early blastula stage; (**K**) mid-blastula stage; (**L**) late blastula stage; (**M**) early gastrula stage; (**N**) mid-gastrula stage; (**O**) late gastrula stage; (**P**) neurula stage; (**Q**) closure of blastopore stage; (**R**) appearance of somite; (**S**) appearance of cerebral vesicle; (**T**) appearance of optic capsule; (**U**) otocyst stage; (**V**) appearance of candal fin; (**W**) muscle effect stage; (**X**) heart-beating stage; (**Y**) early hatching stage; (**Z**) embryo hatching stage.

At 13 min post-fertilization, the single-layered egg membrane transforms into a double-layered structure, and the perivitelline space emerges and gradually expands. At 30 min post-fertilization, the protoplasm begins to flow radially toward the animal pole and accumulates there, while the yolk (oil globules) contracts and sinks to the lower part of the egg. At 1 h post-fertilization, the animal pole bulges to form a dark cap-shaped blastodisc, marking the blastodisc stage (Figure 1A,B).

#### 3.1.2. Cleavage Stage (2 h 09 min–2 h 15 min)

The cleavage stage of *S. glanis* eggs occurs from 1 h to 3 h and 15 min post-fertilization, characterized by partial discoidal cleavage at the blastodisc. At 1 h post-fertilization, the first cleavage initiates. The blastodisc gradually invaginates from the middle of its upper end, forming a cleavage furrow and producing two spherical cells of similar size, defining the two-cell stage (Figure 1C). At 1 h and 27 min post-fertilization, a new cleavage furrow—perpendicular to the first—appears in the middle of the blastodisc’s upper end, forming four uniformly sized and evenly distributed spherical cells, the four-cell stage (Figure 1D). At 1 h and 47 min post-fertilization, the third cleavage commences. Two cleavage furrows, parallel to the first and positioned on either side, form eight evenly distributed, similarly sized spherical cells, the eight-cell stage (Figure 1E). Subsequently, two additional furrows—parallel to the second cleavage and flanking it—produce 16 neatly arranged, uniformly sized spherical cells. The cell volume decreases at this stage, and the cell mass appears rectangular from above, marking the 16-cell stage (Figure 1F). At 2 h and 30 min post-fertilization, 32 neatly arranged, approximately equal-sized spherical cells gradually form. The cell mass remains rectangular in the top view, with significantly smaller cells, indicating the 32-cell stage (Figure 1G). At 2 h and 58 min post-fertilization, cell division becomes asynchronous, generating 64 small spherical cells with size variations, uneven distribution, and irregular arrangement. The cell mass resembles a deformed rectangle from above, the 64-cell stage (Figure 1H). At 3 h and 15 min post-fertilization, cells continue to divide into tiny, irregularly sized spherical cells. The cleavage boundaries blur, and the cell mass concentrates at the animal pole in a hemispherical shape, entering the multicellular stage (Figure 1I).

#### 3.1.3. Blastula Stage (2 h 58 min–3 h 2 min)

The blastula stage occurs from 3 h and 15 min to 6 h and 17 min post-fertilization, divided into early, middle, and late phases. At 4 h post-fertilization, cell numbers increase with more layers of arrangement, and cells shrink in size. Intercellular boundaries become indistinct, forming a highly raised hemispherical blastoderm that protrudes prominently above the yolk mass, the early blastula stage (Figure 1J). At 5 h and 16 min post-fertilization, the blastoderm begins to spread downward from the upper part of the yolk mass, and the blastoderm layer lowers, entering the middle blastula stage (Figure 1K). At 6 h and 17 min post-fertilization, the blastoderm continues to expand downward and thins significantly, covering approximately one-fourth of the yolk mass, marking the late blastula stage (Figure 1L).

#### 3.1.4. Gastrula Stage (7 h 24 min–7 h 26 min)

The gastrula stage occurs from 6 h and 17 min to 13 h and 43 min post-fertilization, including early, middle, and late phases. At 11 h post-fertilization, the blastoderm undergoes epiboly, extending toward the vegetal pole and covering one-third of the yolk mass. The lower edge of the epibolic layer involutes and thickens to form a ring-shaped raised blastoporal lip, defining the early gastrula stage (Figure 1M). At 13 h and 36 min post-fertilization, the epibolic layer continues to extend, covering half the yolk mass. A blastopore emerges beside the blastoporal lip, which develops into the blastopore margin, entering the middle gastrula stage (Figure 1N). At 13 h and 43 min post-fertilization, the blastoderm extends further via epiboly, covering four-fifths of the yolk surface, and the embryonic shield thickens, marking the late gastrula stage (Figure 1O).

#### 3.1.5. Neurula Stage (2 h 22 min–2 h 26 min)

The neurula stage occurs from 13 h 43 min to 16 h 9 min post-fertilization. At 14 h 10 min post-fertilization, the blastoderm continues epiboly, covering five-sixths of the yolk mass. A small, exposed portion of the yolk mass at the vegetal pole forms the yolk plug. The midline of the embryonic shield on the embryo’s dorsal side thickens significantly, entering the stage of neural plate formation and differentiation (Figure 1P). At 16 h 9 min post-fertilization, the blastoderm covers the entire yolk mass, the blastopore closes, and epibolic movement terminates, marking the blastopore closure stage (Figure 1Q).

#### 3.1.6. Organogenesis Stage (27 h 47 min–27 h 51 min)

The organogenesis stage occurs from 16 h 9 min to 44 h post-fertilization. At 17 h post-fertilization, the embryo elongates and encircles half the yolk sac. Two pairs of somites appear, with the head and tail bulging prominently, marking the somite formation stage (Figure 1R). At 17 h 42 min post-fertilization, the anterior end of the embryo protrudes to form an oval cerebral vesicle, with 4–6 pairs of somites present, defining the cerebral vesicle formation stage (Figure 1S). At 24 h 39 min post-fertilization, the embryo encircles three-fourths of the yolk sac. With 14–16 pairs of somites, eye vesicles form on the upper lateral sides of the head, marking the eye vesicle formation stage (Figure 1T). At 28 h 12 min post-fertilization, the embryo encircles four-fifths of the yolk sac. With 22–24 pairs of somites, otic vesicles appear dorsolaterally behind the head. The caudal fin primordium forms and separates from the yolk sac, defining the otic vesicle formation stage (Figure 1U). At 30 h 48 min post-fertilization, the embryo encircles five-sixths of the yolk sac. With 26–30 pairs of somites, maxillary barbel primordia emerge anterolaterally to the eye vesicles. The free portion of the tail elongates, and a caudal fin fold appears, marking the caudal fin formation stage (Figure 1V). At 37 h 9 min post-fertilization, the tail fully detaches from the yolk sac. With 34–38 pairs of somites, dorsal muscle segments exhibit irregular contractions and twists, defining the muscle contraction stage (Figure 1W). At 44 h post-fertilization, a thoracic cavity forms ventral to the embryo’s head, containing a rhythmically beating heart, marking the heart-beating stage (Figure 1X).

#### 3.1.7. Hatching Stage (3 h 49 min–3 h 55 min)

The hatching stage occurs from 44 h to 47 h 55 min of embryonic development. At 46 h 23 min post-fertilization, the egg membrane gradually becomes transparent, thin, and swollen, losing its stickiness and elasticity. The embryo, particularly its tail, twists vigorously, marking the pre-hatching stage (Figure 1Y). By 47 h 55 min of embryonic development, 42–44 pairs of somites are present. The egg membrane near the embryo’s tail ruptures (the rupture serving as the hatching exit), and the embryo continues wriggling until fully emerged, marking the hatching stage (Figure 1Z).

This study is the first to systematically observe that the diameter of hormone-induced fertilized eggs of *S. glanis* after water absorption is (2.88 ± 0.13) mm, with a total accumulated temperature of 1245.56 °C·h for embryonic development. These fundamental data provide a critical baseline for research on the embryonic development of this species. The embryonic mortality rate in the experiment was <3% with no deformed individuals, indicating that the developmental process under (26.0 ± 0.9) °C conditions was stable, and the data have biological representativeness.

### 3.2. Main Characteristics of the Growth and Development of S. glanis Larvae, Juveniles, and Young Fish

The early development of *S. glanis* fry goes through three growth and development stages: the pre-yolk sac larval stage (0–3 days), during which the yolk sac is completely absorbed; the post-larval stage (4–15 days), during which the primordia of various fin rays are formed; the juvenile stage (6–30 days), during which the differentiation of various unpaired fin rays is completed; and the young fish stage (more than 31 days), during which the lateral line is formed, running from the head through to the tail.

#### 3.2.1. Measurable Traits of Larvae, Juveniles, and Young Fish

**Figure 2:**
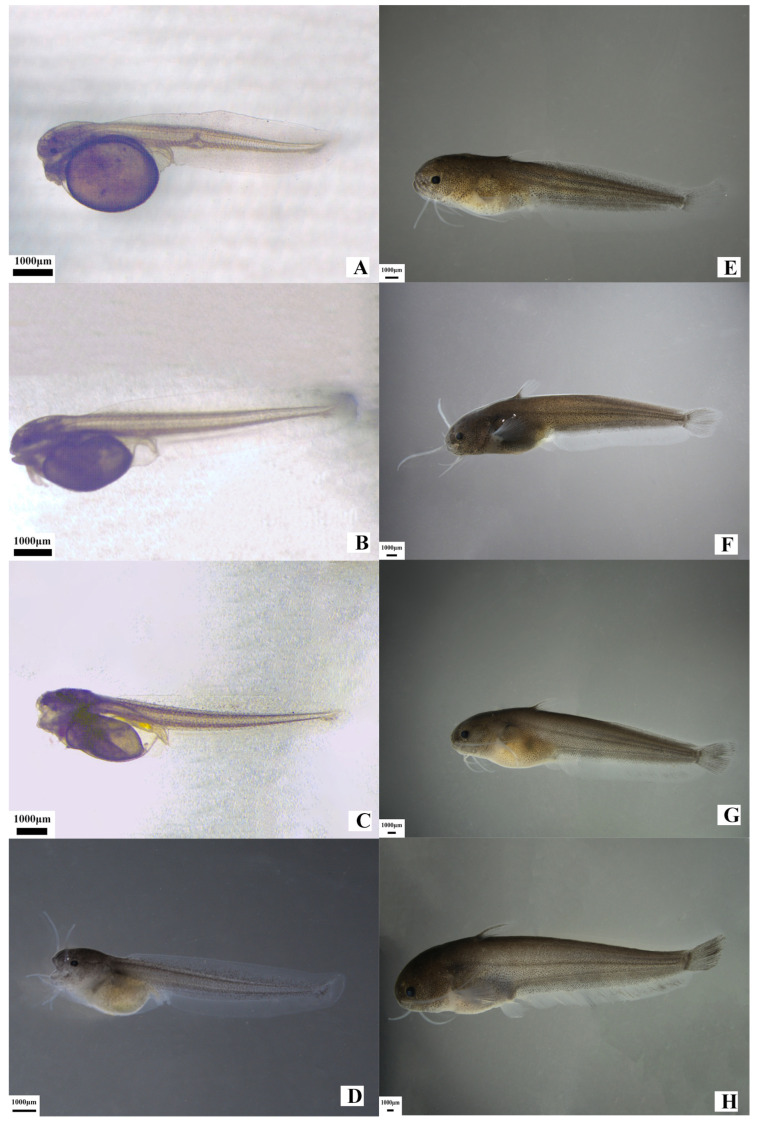
Larva development of *S. glanis* Linnaeus. (**A**) 1 d; (**B**) 2 d; (**C**) 3 d; (**D**) 4 d; (**E**) 15 d; (**F**) 17 d; (**G**) 25 d; (**H**) 30 d.

During the larval, juvenile, and young fish stages of *S. glanis*, the total length, body length, body height, and head length gradually increase with the number of days after hatching (Table 3, Figure 2).

The growth rate of the total length of *S. glanis* larvae and juveniles was slow during the first 20 days, increasing only by (1.28 ± 0.09) mm per day. After 20 days, they began to grow rapidly, with a growth rate of the total length of (1.49 ± 0.11) mm per day. Among them, the body length, body height, and head length showed significant allometric growth trends (Table 3).

#### 3.2.2. Main Describable Traits of Larvae, Juveniles, and Young Fish

① Pre-yolk sac Larval Stage

One-day-old larvae: The head and trunk are laterally compressed, with the gill primordium formed. Melanin in the eye lenses increases, the yolk sac becomes smaller, the intestine thickens, and blood flows in the gill blood vessels (Figure 2A).

Two-day-old larvae: The yolk sac shrinks significantly, with 48–50 pairs of somites present. The upper and lower jaws begin to differentiate, the cerebral cavity expands, the brain is formed, and the chordal nerve extends from the brain to the dorsal region (Figure 2B).

Three-day-old larvae: The yolk sac is almost completely absorbed, and the thoracic and abdominal cavities expand. The heartbeat is distinct, the intestine thickens further, and the primordia of the pectoral, pelvic, anal, dorsal, and caudal fins are formed. The primordium of the second pair of mandibular barbels appears anteromedially to the first pair. At this stage, larvae gain the ability to swim briefly and evade predators (Figure 2C).

② Post-larval Stage

Four-day-old larvae: The yolk sac is almost completely absorbed, the pigmentation on the trunk increases significantly, the fins are initially formed, the intestine becomes thicker and longer, the oral fissure, anus, and cloacal protuberance are formed, the mouth can open and close, and the larvae can float to start feeding on exogenous food (Figure 2D).

From 5 to 14 days old: The anterior part of the intestine expands to form the stomach, the swim bladder is formed above the posterior part of the body cavity, the gills develop and turn red, and its posterior end tapers into a tube connected to the cloacal opening. The heart, especially the red blood continuously circulating in the blood vessels, is clearly visible. The front ends of the upper and lower jaws are basically of the same length, and both the maxillary and mandibular barbels elongate. Fifteen-day-old larvae: Nostrils appear in front of the eyes and on the posterosuperior part of the snout, the body surface pigmentation increases significantly, the black spots expand, and the whole body is almost dark brown. The internal organs are not easy to observe clearly, while the external organs are clearly visible. The fins develop more obviously, the swimming ability is enhanced compared with the previous stage, and the food intake also increases significantly (Figure 2E), marking the end of the post-larval stage.

③ Juvenile Stage

Sixteen-day-old juveniles: The differentiation of various external organs is basically complete, the fins are well-developed, the swimming speed increases, the evasion ability improves, and the feeding behavior becomes more flexible, entering the juvenile stage.

Seventeen-day-old juveniles: The differentiation of various external organs is basically complete, the fins are well-developed, the swimming speed increases, the evasion ability improves, and the feeding behavior becomes more flexible. The mandibular barbels, especially the maxillary barbels, grow significantly, and the lateral line appears on the anterosuperior part of the body, entering the juvenile stage (Figure 2F).

From 18 to 24 days old: The first fin ray of the pectoral fin begins to harden, the lateral line starts to extend backward, the front end of the lower jaw is slightly longer than that of the upper jaw, the fins continue to grow, appear pale and translucent, the first fin ray of the pectoral fin is slender and conical, and the ends of the soft fin rays of each fin begin to branch. Twenty-five-day-old juveniles: The lateral line extends to the caudal peduncle, the body surface increases, the body is smooth, shiny, and yellowish-brown, the nostrils become larger, and there are dark black patterns on both the anal and caudal fins, ending the juvenile development stage (Figure 2G).

④ Young Fish Stage

Thirty-one-day-old young fish: The maxillary barbels are relatively long, the lateral line is complete, the mucus layer on the sides and back of the body thickens, the body is smooth and scaleless, light brown in color, and the abdomen is light white. The lateral line extends to the caudal fin, entering the young fish stage (Figure 2H). From 32 to 40 days old: The feeding ability is enhanced, capable of swallowing relatively large live larvae and even smaller fry. Intraspecific aggression occasionally occurs. They are sensitive to external disturbances, easily find dark environments to hide in, and exhibit morphological characteristics like adult fish.

#### 3.2.3. Changes in the Volume of the Yolk Sac of Larvae

**Figure 3:**
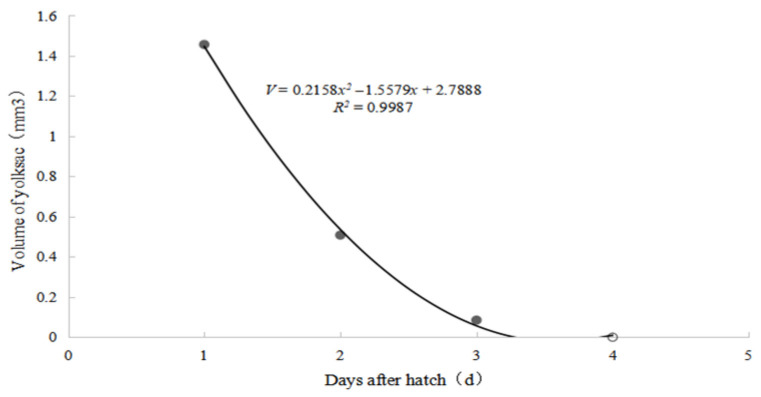
Yolk sac absorption curve of *S. glanis* Linnaeus.

The curve showing the relationship between the volume of the yolk sac of newly hatched *S. glanis* larvae and the number of days after hatching was calculated based on the measured data of the yolk sac diameter, as shown in Figure 3. The relationship between the change in the yolk sac volume and the age of the larvae conforms to the formula *V* = 0.2158*x*^2^ − 1.5579*x* + 2.7888 (*R*^2^ = 0.9987), where *V* represents the volume of the yolk sac and x represents the age in days.

#### 3.2.4. Growth Characteristics of Larvae, Juveniles, and Young Fish

This study is the first to record the initial total length of newly hatched larvae of *S. glanis* as (7.61 ± 0.47) mm. Although there is a lack of comparison with previous data, environmental conditions such as the water temperature and dissolved oxygen were strictly controlled during the experiment (dissolved oxygen 5.35–7.81 mg/L, pH 7.3 ± 0.2), and the fitting degree of the larval growth model was extremely high (total length model (*R*^2^ = 0.9916). This verifies the stability and reliability of the observed data, providing a comparable growth baseline for subsequent studies.

Regression analysis was performed based on the average measured data of the total length, body length, body height, and head length of *S. glanis* larvae, juveniles, and young fish (Table 3). The growth characteristics of the total length, body length, body height, and head length of *S. glanis* during the larval, juvenile, and young fish stages (0–40 days) conform to the following formulas in sequence:

*TL* = 0.0141*x*^2^ + 0.8096*x* + 8.2421(*R*^2^ = 0.9916) (1)

The quadratic term (0.0141*x*^2^) indicates an accelerating growth trend in total length over time, which aligns with the biological characteristic of larvae transitioning from slow initial growth to rapid elongation after 20 days. The positive linear coefficient (0.8096*x*) reflects the baseline daily growth rate, while the constant term (8.2421) approximates the initial total length at hatching.

*BL* = 0.01TE*x*^2^ + 0.5142*x* + 8.9617(*R*^2^ = 0.9894) (2)

Similar to the total length, the quadratic term (0.0112*x*^2^) suggests accelerating body length growth, with a lower coefficient than the total length, indicating that the body length increases at a relatively slower rate compared to the total length—likely due to the tail accounting for an increasing proportion of the total length during development. The linear term (0.5142*x*) and constant term (8.9617) represent the baseline growth rate and initial body length, respectively.

*BH* = 2 *×* 10^−0.6^*x*^5^ + 0.0003*x*^4^ + 0.0096*x*^3^ + 0.1536*x*^2^ + 0.8193*x* − 3.3467 (*R*^2^ = 0.9803) (3)

The fifth-order polynomial was selected for body height (BH) to capture its complex, multi-phasic growth patterns. Biological observations indicate that body height growth is non-monotonic: rapid thickening occurs in the early stages (1–10 days) as visceral organs develop, followed by a temporary slowdown (10–20 days) when energy is prioritized for longitudinal growth, and a second phase of moderate thickening (20–40 days) as muscle and skeletal structures mature. Lower-order models (linear, quadratic, or cubic) were tested but failed to accurately reflect these inflection points: linear models (*R*^2^ = 0.82) could not account for growth acceleration/deceleration, while cubic models (*R*^2^ = 0.93) underestimated the late-stage thickening. The small coefficients of high-order terms (e.g., 2 *×* 10^−0.6^*x*^5^) indicate they primarily refine local inflections rather than driving overall trends, ensuring biological plausibility.

*HL* = 0.0002*x*^3^ + 0.0106*x*^3^ + 0.385*x* + 0.8476(*R*^2^ = 0.9840) (4)

The cubic term (0.0002*x*^3^) reflects the changing growth rate of the head length: rapid initial growth (1–15 days) as sensory organs (eyes, barbels) and cranial bones develop, followed by a gradual slowdown—consistent with the head accounting for a decreasing proportion of the total length in later stages. The quadratic term (0.0106*x*^2^) and linear term (0.385*x*) further modulate the growth trajectory, while the constant term (0.8476) approximates the initial head length at hatching.

In Formulas (1), (2), (3), and (4), TL represents the total length, BL represents the body length, BH represents the body height, HL represents the head length, and *x* represents the age in days.

The growth models revealed distinct allometric patterns in *S. glanis*’ early development. The total length model (*R*^2^ = 0.9916) showed a growth acceleration after 20 days (1.49 ± 0.11 mm/day vs. 1.28 ± 0.09 mm/day before 20 days), which is more pronounced than in closely related species such as *Silurus asotus* (1.12 mm/day) [25] and *Pangasianodon hypophthalmus* (1.05 mm/day) [23]. This difference may relate to *S. glanis*’ carnivorous adaptation, where rapid post-feeding growth enhances predatory competitiveness.

The quadratic term (0.0141*x*^2^) in the total length model indicates accelerating growth driven by skeletal ossification and muscle development, while the linear term (0.8096*x*) reflects baseline growth supported by yolk and exogenous nutrition. In contrast, the fifth-order polynomial for body height (*R*^2^ = 0.9803) captures complex morphological adjustments during fin differentiation, which is absent in the linear growth model of *Clarias gariepinus* (*TL* = 0.56*x* + 5.2). Head length growth (cubic model, *R*^2^ = 0.9840) showed the early prioritization of sensory organ development, with barbels reaching 40% of head length by 15 days, a trait critical for foraging in benthic environments—consistent with specialized predatory strategies in Siluriformes.

## 4. Discussion

### 4.1. Biological Adaptability of Embryonic Development Timing in S. glanis

This study found that the entire embryonic development of European wels catfish (*S. glanis*) takes 47 h and 55 min, with an accumulated temperature of 1245.56 °C·h. This development duration and accumulated temperature characteristics form a precise ecological adaptation to its freshwater benthic lifestyle. The mature eggs of *S. glanis* are yellow and spherical, sticky, with an average egg diameter of (2.23 ± 0.12) mm; the average diameter of fertilized eggs is (2.35 ± 0.11) mm, which rapidly swells to (2.88 ± 0.13) mm after water absorption, and the total length of newly hatched larvae is (7.61 ± 0.47) mm. Compared with non-siluriform fish, the fertilized eggs of *Onychostoma rara* [26] swell to (2.88 ± 0.07) mm after water absorption, and the total length of hatched larvae is (6.67 ± 0.53) mm; the fertilized eggs of freshwater drum (*Aplodinotus grunniens*) [27] are only (1.07 ± 0.04) mm, and (1.41 ± 0.03) mm after water absorption, with a larvae total length of 2.73–3.10 mm. The egg diameter and larval total length of *S. glanis* are 2.08 times and 2.61 times those of *A. grunniens*, respectively, demonstrating the advantages of a large-egg reproduction strategy. Compared with close relatives in *Siluriformes, Silurus asotus* [25] has an egg diameter of 1.53 mm and larval total length of 4.8 mm; *Pangasianodon hypophthalmus* [23] has an average egg diameter of (1.60 ± 0.23) mm, and the total length of hatched larvae is (4.38 ± 0.23) mm; and *Clarias gariepinus* [28] has an egg diameter of 1.7–1.9 mm and larval total length of 2.30 mm (Table 4). The eggs of *S. glanis* are significantly larger, and the hatched larvae are longer, showing a remarkable size advantage in Siluriformes.

From the perspective of reproductive biology mechanisms, fish egg diameter is a core indicator reflecting egg quality, which is significantly positively correlated with the fertilization rate, hatching rate, and larval survival rate [29]. The egg diameter of *S. glanis* reaches 2.88 mm after water absorption, corresponding to a yolk volume of 3.98 mm^3^, which is 3.29 times that of *C. gariepinus* (1.21 mm^3^), providing sufficient energy reserves for embryonic development. Meanwhile, the total length of hatched larvae is 7.61 mm, with an initial swimming speed of 0.8 cm/s, significantly higher than that of *A. grunniens* (0.3 cm/s), enhancing the ability to avoid predators [30]. This “large-egg–large-larva” strategy enables *S. glanis* to have higher reproductive success and juvenile survival rates in benthic environments.

The period from the neurula stage to the hatching stage in *S. glanis* embryo development accounts for only 21.3% of the total duration, showing a “rapid progression” characteristic, which may be a stress adaptation to freshwater predators. When the water temperature fluctuates, the intense twisting behavior observed 2 h before the tail bud breaks through the egg membrane contrasts sharply with the passive hatching mode of cyprinid fish. This active hatching mechanism can reduce the embryonic retention time and lower the risk of Saprolegnia infection [31]. Furthermore, the observed 42–44 somite pairs lay a myotome foundation for juvenile cruising ability, confirming the evolutionary logic of “structural pre-adaptation” [32].

### 4.2. Morphogenesis and Functional Differentiation During Larval and Juvenile Development

The early development of European wels catfish (*S. glanis*) exhibits typical allometric growth characteristics, with its growth kinetics precisely spatiotemporally coupled to the morphological differentiation of organ systems. In the first 20 days after hatching, the daily growth rate of the total length is 1.28 ± 0.09 mm, and after 20 days of age, it surges to 1.49 ± 0.11 mm (*p* < 0.01). This growth inflection point is highly synchronized with the morphological transformation of the digestive system [33]. During the yolk sac state (0–3 days old), the digestive tract is a short straight tube (is 1.12 ± 0.08 mm), matching the absorption process of yolk substances; in the late larval stage (4–15 days old), with the formation of intestinal spiral valves (16 ± 2 valves) and significant expansion of the mouth fissure (fissure width 0.58 ± 0.04 mm), the key transition from endogenous to exogenous feeding is gradually completed [34].

**Figure 4:**
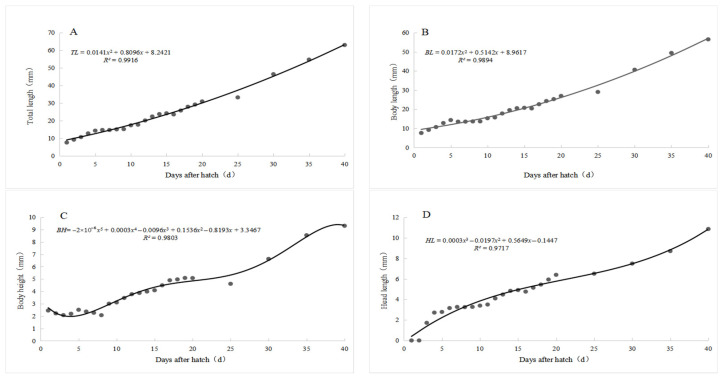
The early growth curve of *S. glanis* Linnaeus. (**A**) Represents the relationship between total length and day age of *S. glanis* Linnaeus larvae. (**B**) Represents the relationship between body length and day age of *S. glanis* Linnaeus larvae; (**C**) Represents the relationship between body height and day age of *S. glanis* Linnaeus larvae. (**D**) Represents the relationship between head length and day age of *S. glanis* Linnaeus larvae.

There is a deep synergistic mechanism between the transformation of growth strategies and the development of predatory adaptability. As an apex predator in freshwater ecosystems, *S. glanis* larvae already possess remarkable predatory specialized structures: the primordium of the mandibular barbels appears at 3 days old, and the barbel length reaches 1.82 ± 0.15 mm (accounting for 40% of head length) at 15 days old. The growth acceleration phenomenon after 20 days old (Figure 4A), from the larva to juvenile and young fish stages, is caused by reasons like the allometric growth strategies of other fish [35,36], continuing until the end of the juvenile stage and entry into the young fish stage.

The specialization of sensory organs and ecological adaptation strategies in 30-day-old juvenile *S. glanis* have been revealed in multiple studies. The black markings on the dorsal and caudal fins exhibit special optical properties, highly matching the reflectivity of the substrate environment. This matching may enable individual recognition through intraspecific visual signals (such as synchronous movement during group aggregation) or achieve camouflage adaptation by mimicking the substrate environment [37]. This evolutionary pattern is representative of Siluriformes. For example, although the lateral line system of Ictalurus punctatus is also well-developed, there are differences in the distribution of neuromasts and types of mechanoreceptors [38]; while the lateral line development of *Clarias gariepinus* is dominated by free neuromasts, contrasting with the canal neuromast structure of *S. glanis* [39].

### 4.3. Ecological Significance of Growth Models and Enlightenment for Aquaculture Practices

In the development process of larvae, juveniles, and young fish of *S. glanis*, the established growth regression model shows a significant quadratic curve characteristic (*TL* = 0.0141*x*^2^ + 0.8096*x* + 8.2421, *R*^2^ = 0.9916), contrasting with the linear growth pattern of *Clarias gariepinus* (*TL* = 0.56*x* + 5.2) [40,41]. This growth model has important application values in aquaculture: The hatching time can be accurately predicted through the accumulated temperature model. Under the water temperature of 25 °C, the theoretical hatching time is 49 h (prediction error ± 1.2 h) [42]; taking the growth inflection point at 20 days old as the boundary, it is recommended to adjust the feeding strategy—introducing large cladocerans (such as Daphnia magna) at this stage can increase the larval survival rate to 82.3%, which is 37.5% higher than conventional feeding [43].

The yolk sac absorption curve of *S. glanis* (*V* = 0.2158*x*^2^ − 1.5579*x* + 2.7888, *R*^2^ = 0.9765) presents a unique trend of “fast first, then slow”, with the yolk sac volume decreasing by 68.2% at 3 days old, which is highly consistent with the critical period of intestinal differentiation (48–72 h post fertilization) [44,45]. This finding suggests that precise feeding of first feeding should be completed at 3 days old in artificial seedling rearing to avoid nutritional gaps. In terms of trunk development, the quintic polynomial model of body height growth (*R*^2^ = 0.9803) reveals the complexity of dorsal fin and adipose fin differentiation [46], providing a morphological basis for seedling grading cultivation—when the body height growth rate shows a stage-specific decrease (such as at 35 days old), it can be used as the optimal timing for grading and screening [47]. Therefore, it is recommended to timely separate the ponds during the juvenile stage (20–30 days old), control the stocking density below 50 individuals/L, and ensure sufficient feed through precise feeding to effectively reduce the risk of cannibalism [48].

This study did not include a natural spawning control group primarily due to the inability of *S. glanis* to spawn naturally in pond culture environments. Despite simulating natural conditions such as the water temperature (26.0 ± 0.9 °C) and substrate, the broodstock exhibited no natural spawning behavior, making it impossible to obtain embryos or larvae from natural reproduction. Introducing control groups from non-pond environments (e.g., laboratories) would have created significant discrepancies in living conditions compared to the pond system used in this study, violating the “single variable” principle and invalidating the comparative results. Although the hormone-induced group showed low embryonic mortality (<3%), no deformities, and key indicators (e.g., egg diameter 2.88 ± 0.13 mm, hatching time 47 h 55 min) consistent with reported data for natural development under similar temperatures, potential effects of hormone treatment on growth models cannot be fully excluded. Future studies will optimize pond environments to induce natural spawning or establish non-treated control groups under the same conditions to verify hormone impacts on growth dynamics.

## 5. Conclusions

This study systematically observed the morphological development of embryos and larval–juvenile stages of *S. glanis*. The results showed that the fertilized eggs are yellow, sticky, and spherical, with a diameter of (2.88 ± 0.13) mm after water absorption. Embryonic development goes through seven stages and 26 periods, taking a total of 47 h and 55 min at a water temperature of (26.0 ± 0.9) °C, with a total accumulated temperature of 1245.56 °C·h. The development of larval–juveniles can be divided into the pre-yolk sac larval stage, late larval stage, juvenile stage, and young fish stage, and a total length growth model was established as *TL* = 0.0141*x*^2^ + 0.8096*x* + 8.2421 (*R*^2^ = 0.9916). These findings not only provide a scientific basis for the division of early developmental stages and reproductive seedling cultivation of *S. glanis*, but the revealed biological characteristics such as embryonic development timing and body growth strategies can also offer fundamental references for ecological conservation plans of its native habitats (e.g., habitat restoration, optimization of the timing for stock enhancement and release), contributing to the sustainable maintenance of the natural populations of this species.

## Figures and Tables

**Table 1:** Temperatures of *S. glanis* Linnaeus at different stages of embryonic development.

Development Stage	Elapsed Time	Water Temperature (°C)	Accumulative Temperature (°C·h)
Zygophase stage	56 min–1 h 0 min	26.0 ± 0.9	26.0
Cleavage stage	2 h 09 min–2 h 15 min	26.0 ± 0.9	58.50
Blastula stage	2 h 58 min–3 h 2 min	26.0 ± 0.9	78.78
Gastrula stage	7 h 24 min–7 h 26 min	26.0 ± 0.9	193.18
Neurula stage	2 h 22 min–2 h 26 min	26.0 ± 0.9	63.18
Organogenesis stage	27 h 47 min–27 h 51 min	26.0 ± 0.9	724.10
Hatching stage	3 h 49 min–3 h 55 min	26.0 ± 0.9	101.82
Total	47 h 27 min–47 h 55 min	26.0 ± 0.9	1245.56

**Table 2:** Embryonic development of *S. glanis* Linnaeus.

Embryonic Development Stage	Time After Fertilization	Developmental Characteristics	Figure 1
Fertilized egg	0	Fertilized eggs are spherical demersal eggs, yellow in color, with uniformly distributed egg cytoplasm. Upon water absorption and swelling, a double-layered egg membrane is formed, where the outer membrane exhibits slight adhesiveness.	A
Blastodisc stage	13 min	The non-yolk cytoplasm separates and flows toward the animal pole, leading to the formation of the blastodisc.	B
2-cell stage	1 h	A meridional cleavage furrow appears in the center of the blastodisc, dividing it into two blastomeres of equal size.	C
4-cell stage	1 h 27 min	A cleavage furrow perpendicular to the first one forms, giving rise to four equally sized cleavage cells arranged in a 2 × 2 pattern.	D
8-cell stage	1 h 47 min	Three cleavage furrows perpendicular to the second cleavage furrow appear, resulting in eight cleavage cells arranged in a 2 × 4 pattern.	E
16-cell stage	2 h 12 min	Two cleavage furrows parallel to the second cleavage furrow form on both sides of the second cleavage furrow, resulting in sixteen cleavage cells arranged in a 4 × 4 pattern.	F
32-cell stage	2 h 30 min	Thirty-two cleavage cells of similar size are arranged in a 4 × 8 pattern.	G
64-cell stage	2 h 58 min	Sixty-four blastomeres are formed, with individual cells becoming smaller, showing size differences, and uneven distribution.	H
Multicellular stage	3 h 15 min	The dividing cells increase in number and become more densely packed, appearing smaller in size and varying in dimension with blurred boundaries, thus forming an overlapping, elevated cell mass concentrated at the animal pole (upper end of the yolk mass).	I
Early blastula stage	4 h	The cells continue to divide, forming a highly elevated, hemispherical blastoderm that towers above the upper end of the yolk mass.	J
Mid-blastula stage	5 h 16 min	The blastoderm begins to spread downward from the upper part of the yolk mass, and the germ layer starts to lower and envelop the upper portion of the yolk sac.	K
Late blastula stage	6 h 17 min	The blastoderm expands toward the vegetal pole and 明显 thins, covering approximately one-quarter of the yolk mass.	L
Early gastrula stage	11 h	The germ layer envelops one-third of the yolk sac, and the lower edge of the enveloping layer thickens to form a circular, elevated germ ring.	M
Mid-gastrula stage	13 h 36 min	The germ layer continues to extend downward, enveloping half of the yolk mass, and the blastopore and embryonic shield are formed.	N
Late gastrula stage	13 h 43 min	The blastoderm continues to epibolize, covering four-fifths of the yolk sac, and the embryonic shield thickens.	O
Neurula stage	14 h 10 min	The germ layer envelops five-sixths of the yolk mass, with a neural plate forming on the embryonic back and a yolk plug developing at the vegetal pole.	P
Closure of blastopore stage	16 h 9 min	The germ layer completely covers the yolk, the blastopore closes, and the epibolic movement ends.	Q
Appearance of somite	17 h	The embryo elongates, encircling half of the yolk sac. The head and tail regions bulge prominently, and two pairs of somites appear in the trunk.	R
Appearance of cerebral vesicle	17 h 42 min	The embryonic trunk, including the head region, develops brain vesicles and 4 to 6 pairs of somites.	S
Appearance of optic capsule	24 h 39 min	The embryo encircles three-quarters of the yolk sac, with 14–16 pairs of somites. Optic vesicles emerge dorsolaterally on both sides of the head.	T
Otocyst stage	28 h 12 min	The embryo encircles four-fifths of the yolk sac, featuring 22–24 pairs of somites. Otic vesicles appear dorsolaterally behind the head.	U
Appearance of candal fin	30 h 48 min	The embryo encircles five-sixths of the yolk sac, with 26–30 pairs of somites. The free part of the tail lengthens, and the caudal fin fold appears.	V
Muscle effect stage	37 h 9 min	The embryo encircles five-sixths of the yolk sac, with 26–30 pairs of somites. The free portion of the tail elongates, and the caudal fin fold appears.	W
Heart-beating stage	44 h	A ventricular cavity (thoracic cavity) forms beneath the ventral side of the embryonic head, and a rhythmically beating heart appears within the cavity.	X
Early hatching stage	46 h 23 min	The embryonic membrane gradually becomes transparent, thin, and loses its viscosity. The number of somites reaches 42–44 pairs, and the embryo—especially the tail—twists vigorously.	Y
Embryo hatching stage	47 h 55 min	The embryo hatches out of the membrane. Ni	Z

**Table 3:** Data of main measurable traits during the growth and development of larvae and juveniles of *S. glanis* Linnaeus.

Developmental Period	Days After Hatch/d	Total Length/mm	Body Length/mm	Body Height/mm	Head Length/mm
Pre-yolk sac larval stage	1	7.61 ± 0.47	7.61 ± 0.47	2.45 ± 0.10	1.12 ± 0.03
	2	9.22 ± 0.54	9.22 ± 0.54	2.22 ± 0.10	1.46 ± 0.09
	3	10.71 ± 0.25	10.71 ± 0.25	2.08 ± 0.11	1.72 ± 0.01
Larvae anaphase	4	12.80 ± 0.27	12.80 ± 0.27	2.19 ± 0.06	2.72 ± 0.06
	5	14.36 ± 0.45	14.36 ± 0.45	2.51 ± 0.11	2.78 ± 0.11
	6	14.75 ± 0.43	13.52 ± 0.48	2.37 ± 0.15	3.16 ± 0.07
	7	14.72 ± 0.33	13.54 ± 0.32	2.27 ± 0.06	3.26 ± 0.07
	8	15.09 ± 0.66	13.64 ± 0.63	2.08 ± 0.14	3.25 ± 0.17
	9	15.21 ± 0.96	13.66 ± 0.81	3.01 ± 0.14	3.27 ± 0.14
	10	17.39 ± 1.24	15.32 ± 0.99	3.11 ± 0.12	3.40 ± 0.10
	11	17.75 ± 0.70	15.73 ± 0.62	3.47 ± 0.16	3.50 ± 0.15
	12	20.14 ± 0.92	17.79 ± 0.76	3.78 ± 0.12	4.10 ± 0.18
	13	22.40 ± 0.63	19.54 ± 0.60	3.88 ± 0.12	4.48 ± 0.16
	14	23.77 ± 1.09	20.56 ± 1.00	3.99 ± 0.11	4.84 ± 0.14
	15	24.21 ± 0.88	20.75 ± 0.79	4.09 ± 0.18	4.91 ± 0.11
Advanced fry stage	16	23.58 ± 0.92	20.44 ± 0.77	4.49 ± 0.41	4.76 ± 0.44
	17	25.79 ± 1.54	22.63 ± 1.36	4.90 ± 0.29	5.14 ± 0.27
	18	27.93 ± 1.30	24.30 ± 1.05	4.97 ± 0.32	5.45 ± 0.30
	19	29.19 ± 0.77	25.29 ± 0.67	5.09 ± 0.23	5.94 ± 0.14
	20	30.96 ± 1.70	26.97 ± 1.55	5.08 ± 0.24	6.40 ± 0.53
	25	33.24 ± 1.85	29.04 ± 1.70	4.62 ± 0.30	6.51 ± 0.20
	30	46.43 ± 4.09	40.65 ± 3.90	6.63 ± 0.23	7.49 ± 0.12
Juvenile stage	35	54.70 ± 1.31	49.42 ± 1.23	8.55 ± 0.31	8.71 ± 0.38
	40	63.01 ± 0.71	56.52 ± 0.74	9.31 ± 0.24	10.86 ± 0.55
ANOVA (*f*/*p*)		1209.23/*p* < 0.01	1087.71/*p* < 0.01	912.28/*p* < 0.01	934.82/*p* < 0.01

**Table 4:** Comparison of egg diameter and larval total length characteristics between *S. glanis* and other fish species.

Fish Species	Egg Diameter After Water Absorption (mm)	Body Length at Hatching (mm)	References
*S. glaris*	2.88 ± 0.13	7.61 ± 0.47	This study
*Onychostoma rara*	2.88 ± 0.07	6.67 ± 0.53	[26]
*Aplodinotus grunniens*	1.41 ± 0.03	2.73–3.10	[27]
*Silurus asotus*	1.53	4.8	[25]
*Pangasianodon hypophthalmus*	1. 60 ± 0. 23	4.38 ± 0.23	[23]
*Clarias gariepinus*	1.7–1.9	2.30	[28]

## Data Availability

Because the project is not finalized, a link to the data has not been made public.
